# Twelve‐month prevalence, persistence, severity, and treatment of mood and anxiety disorders in Qatar's national mental health study

**DOI:** 10.1002/mpr.2012

**Published:** 2024-05-10

**Authors:** Salma M. Khaled, Sheik Mohammed Al‐Thani, Nancy A. Sampson, Ronald C. Kessler, Peter W. Woodruff, Majid Alabdulla

**Affiliations:** ^1^ Department of Population Medicine College of Medicine Qatar University Doha Qatar; ^2^ Department of Public Health Ministry of Public Health Doha Qatar; ^3^ Department of Health Care Policy Harvard Medical School Boston Massachusetts USA; ^4^ School of Medicine and Population Health University of Sheffield Sheffield UK; ^5^ Hamad Medical Corporation Qatar Hamad Medical Corporation Doha Qatar; ^6^ College of Medicine Qatar University Doha Qatar

**Keywords:** 12‐month prevalence, anxiety disorder, mood disorder, treatment adequacy

## Abstract

**Objectives:**

To estimate 12‐month prevalence, persistence, severity, and treatment of mental disorders and socio‐demographic correlates in Qatar.

**Methods:**

We conducted the first national population‐based telephone survey of Arab adults between 2019 and 2022 using the Composite International Diagnostic Interview and estimated 12‐month DSM‐5 mood and anxiety disorders and their persistence (the proportion of lifetime cases who continue to meet 12‐month criteria).

**Results:**

The 12‐month prevalence of any disorder was 21.1% (10.4% mild, 38.7% moderate, and 50.9% severe) and was associated with: younger age, female, previously married, and with persistence of any disorder. Persistence was 74.7% (64.0% mood and 75.6% anxiety) and was significantly associated with secondary education or lower. Minimally adequate treatment received among those with any 12‐month mental disorder was 10.6% (74.6% in healthcare and 64.6% non‐healthcare sectors). Severity and the number of disorders significantly associated with each other and with treatment received (*χ* ^2^ = 7.24, *p = *0.027) including adequate treatment within the mental health specialty sector (*χ* ^2^ = 21.42, *p < *0.001).

**Conclusions:**

Multimorbidity and sociodemographics were associated with 12‐month mental disorder. Treatment adequacy in Qatar are comparable to high‐income countries. Low treatment contact indicate need for population‐wide mental health literacy programes in addition to more accessible and effective mental health services.

## 1 INTRODUCTION

The burden of disease in epidemiology is typically considered in terms of prevalence (Rothman, 2012), which is defined as the proportion of the population with a condition at a specific point in time (point prevalence) or during a period of time (period prevalence). Worldwide, mental disorders are burdensome conditions accounting for much years lived with disability (YLDs) and years of life lost due to premature mortality (YLLs) because of prevalent cases in a population. Prevalence is also a useful concept as it relates directly to incidence or risk as well as duration of disease (Rothman, 2012). Therefore, death, treatment or recovery from a mental disorder or the lack of thereof can influence the prevalence making it possible to monitor changes in distribution of a mental disorder in the population.

Information about period prevalence especially over the 12‐month period (or 12‐month prevalence) typically considered for policy planning purposes is more useful for policy‐makers than information about prevalence for other longer time periods (e.g. lifetime prevalence). Prevalence estimates over longer recall periods also are more susceptible to recall bias than estimates over shorter recall periods (Regier et al., 1998). Additionally, 12‐month prevalence of a mental disorder is less likely to be underestimated due to selection bias (due to those who are currently suffering from a mental disorder are less likely to participate in research) and is more actionable from public policy perspective than shorter recall periods (e.g. 2‐weeks prevalence). Compared to point‐prevalence or current symptomology (at the time of the interview), 12‐month prevalence estimates are also less susceptible to overestimation due to self‐resolving symptoms or natural fluctuation of mood and anxiety symptoms in response to daily stressors and life events.

Twelve‐month prevalence estimates are context‐specific often influenced by policy and mental service availability, access, and utilization at the country level. The 12‐month prevalence estimates of common mental disorders have been widely reported within the World Mental Health (WMH) consortium comprising some of the largest number of epidemiological studies conducted to date (Kessler & Üstün, 2004). However, only few of these studies were conducted in the Eastern Mediterranean Region (EMR) – a World Health Organization (WHO) – defined as a group of countries with diverse economies and political situations ranging from low‐, middle‐, and high‐ income countries as well as war‐afflicted and politically stable countries. Common and unique cultural challenges beyond the immediate vices of war face women and men in some of these countries including traditional gender roles (Lari, 2023), polygamy (Shepard, 2013), intimate partner violence (Al‐Ghanim, 2009), and socioeconomic inequalities related to nationality (Khaled et al., 2016), sectarianism (Gengler, 2020), and migration (Khaled, 2019; Khaled & Gray, 2019).

Twelve‐month estimates from three WMH studies conducted in the EMR countries (Lebanon, Iraq, and Saudi Arabia) show that 12‐month prevalence of any mental disorder range from 13.6% in Iraq (Alhasnawi et al., 2009), 17.0% in Lebanon (Karam et al., 2006), and 20.2% in Saudi Arabia (Altwaijri, Al‐Subaie, et al., 2020). All three studies reported low treatment among those with 12‐month disorder ranging from 11% to 14% and showed that disorder severity was associated with receiving any 12‐months treatment (Alhasnawi et al., 2009; Altwaijri, Al‐Subaie, et al., 2020; Karam et al., 2006).

To date, there are no data on the current burden and treatment of common mental disorders in the general population of Qatar. The only available data are extrapolations from the Institute for Health Metrics and Evaluation (2019) in which the DALYS for depressive, anxiety, and bipolar disorders ranked first, second, and third, respectively (Global Health Estimates, 2019). Additionally, few limited studies reported on prevalence estimates, correlates of depression, and generalized anxiety in the past 2 weeks (Khaled, 2019; Khaled & Gray, 2019; Khaled & Zolezzi, 2021). The World Mental Health Qatar (WMHQ) is the first population‐based epidemiological study of common mental disorders in the country with the aim to report on the 12‐month prevalence, persistence, severity, treatment, and correlates of common mental disorders in the Arab population of Qatar.

## 2 METHODS

### 2.1 Sample

Sampling design and procedures were described in details earlier in this issue (Khaled, Amro, Bader, et al., 2023). A national‐level cellular telephone frame was used to draw a disproportionate stratified representative sample of Arabic‐speaking adults 18 years or older who lived in Qatar during the survey reference period (January 2019 and January 2022). Potential participants were contacted with a Short Message Service (SMS) text prior to being phoned for an interview using a remote Computer Assisted Telephone Interviewing (CATI) system. The data collection period spanned 234 days during which a total of 5195 interviews were completed in three data collection waves (pilot, wave I, and wave II). All interviews were conducted in Arabic. The average length of the survey was 77 min (63.2 to 94.0). Approximately 72% non‐Qataris Arabs and 28% Qataris completed the interview. The response rate varied with the data collection wave from 17% to 26% with an overall average of approximately 19%. Details about length of interview and response rate calculations were previously described earlier in this issue (Khaled, Amro, Bader, et al., 2023).

#### 2.1.1 Field procedures

Survey field training, administration, and quality control are described in detail in an earlier manuscript in this issue (Khaled, Amro, Bader, et al., 2023).

#### 2.1.2 Ethics

Qatar University's ethics committee approved the survey questionnaire (QU‐IRB 1219‐EA/20). Before beginning the telephone interview, verbal consent was obtained from each eligible subject who wished to participate in the study using a consent script that was included as part of the introduction section in the survey's interview instrument. Interviewers explained survey procedures as well as study aims verbally to the participants over the phone including their right to stop or withdraw from the study at any point during the interview.

#### 2.1.3 Short‐ and long‐ interview

The survey was designed as short‐ and long‐ forms (part I and part II). Using an algorithm where we divided all respondents into one of three groups at the end of the short interview (Part I) for the WMHQ instrument: (1) those who we considered to have “threshold” disorders, (2) those who had “sub‐threshold disorders” and (3) all others. The algorithm roughly selected 100% of the people in the first group, 50% of those in the second group, and 25% of the people in the third group into the long form (Part II). Weights were added to the final dataset to adjust for the under‐sampling of respondents in the second and third groups so that the weighted prevalence of disorders in the final sample had the same expected value as in the total original sample.

### 2.2 Measures

#### 2.2.1 Demographic variables

Sociodemographic correlates included age at interview (defined by the following categories: 18–29 years, 30–39 years, 40–49 years, and 50+ years), gender, and marital status (currently married, previously married, and never married). Highest education achieved by age at interview was defined as low (none, primary or preparatory school), low‐average (secondary school), high‐average (2‐year diploma), and high (bachelor's degree or more).

#### 2.2.2 Twelve‐month prevalence and persistence of mental disorders

Previous WMH surveys used the WHO Composite International Diagnostic Interview (CIDI) version 3.0 (Kessler & Üstün, 2004); we used an updated version of the questionnaire, the WHO CIDI‐3.3, with diagnostic criteria based on the Diagnostic and Statistical Manual of Mental Disorders, 5th Edition or DSM‐5 (American Psychiatric Association, 2013). Twelve‐month disorders considered here included anxiety disorders such as panic disorder (PD) and generalized anxiety disorder (GAD), post‐traumatic stress disorder (PTSD), and obsessive‐compulsive disorder (OCD) and mood disorders including Major Depressive Disorder (MDD) and broad bipolar disorder I/II (BPD). PTSD and OCD were both assessed in part II of the survey. Although PTSD and OCD appear as separate disorder categories in the DSM‐5, we grouped them under anxiety disorder in our analysis in line with their traditional classification in the previous version of the DSM. Comorbid disorders were defined as the number of disorders, exactly 1, exactly 2, and 3 or more. Persistence was defined as the ratio of 12‐month prevalence to lifetime prevalence.

#### 2.2.3 Mental disorder severity

Respondents were categorized as having a serious or severe mental illness if they: (1) met criteria for 12‐month BPD, or (2) met criteria for one or more 12‐month disorder (MDD, GAD, PD, PTSD, OCD) and had either very high 12‐month disorder‐related interference or high or very high 30‐day interference in two or more areas of life (home, work, relationships, or social life), or (3) met criteria for 12‐month MDD, GAD, or PTSD and had very high 30‐day disorder‐related distress or interference. Among those who were not categorized as severe, respondents were labeled moderate if they: (1) met criteria for at least one 12‐month disorder and had either moderate or high 12‐month disorder‐related interference or moderate, high, or very high 30‐day interference in one or more areas of life, or (2) met criteria for 12‐month MDD, GAD, or PTSD and had moderate or high 30‐day disorder‐related interference. The remaining respondents with any 12‐month disorder were categorized as mild.

#### 2.2.4 Twelve‐month treatment

All respondents who completed part II of the survey were asked about ever receiving treatments for problems with emotions, nerves, or mental health. Treatment variables were created using questions detailing the services received from specific providers, which included Mental Health Hospitalization (MHH: hospitalized overnight for problems with emotions, nerves, or mental health), Mental Health Specialist (MHS: psychiatrist, psychologist, mental health counselor, social worker, marriage or family counselor, or psychiatric nurse), General Medical Professional (GMP: general medical doctor, nurse, or other general medical care provider), Spiritual Advisor (SA: Imam, minister, priest, healer, or other spiritual advisor), and Complementary and Alternative Medicine (CAM; self‐help or support group not lead by a mental health professional). Service providers were then collapsed into the following groups to capture specific treatment sectors: Any Healthcare Services (Mental Health Hospitalization or, Mental Health Specialist, or General Medical Professional), Non‐Healthcare Services (Spiritual Advisor or CAM), and Any Treatment Sector (Healthcare and Non‐Healthcare).

#### 2.2.5 Treatment adequacy

We adopted the same definition of treatment adequacy in our analysis, as did other WMH surveys. We defined minimally adequate treatment as four or more visits in past 12 months to any service provider; or two or more visits in past 12 months to any service provider and prescribed any mental health medication in the past 12 months; or still in treatment for a mental health problem.

## 3 ANALYTICAL PROCEDURES

### 3.1 Twelve‐month prevalence analysis

Sociodemographic correlates of 12‐month prevalence and persistence were examined using logistic regression. The models for prevalence were estimated in the total sample. The models for persistence, were estimated by focusing on the subsamples of respondents with the lifetime disorders and predicting 12‐month prevalence controlling for time‐since‐onset (TSO), where TSO was defined as the difference between age‐at interview and age‐of‐onset (AOO). AOO was not included in the model because age at interview was one of the predictors and AOO is perfectly defined by age at interview minus TSO. We estimated separate multivariate models to predict 12‐month prevalence and 12‐month persistence of each disorder assessed in the survey along with parallel models to predict prevalence and persistence of at least one disorder in each of the two broad disorder categories (anxiety, mood) and any disorder. The models for persistence within categories and for any disorder controlled for TSO of the disorder with the most recent AOO in the category as well as for total number of lifetime disorders in the category. Standard errors of estimates of prevalence, persistence, and the logits in the prediction models were obtained using the Taylor series linearization method (Wolter, 1985). Implemented in SAS software version 9.4 (SAS Institute, 2016). Logits and logits ±2 standard errors were exponentiated to produce ORs and 95% CIs. Multivariate significance tests of predictor sets were made with Wald χ2 tests using Taylor series design‐based coefficient variance–covariance matrices. Statistical significance was evaluated consistently at the 0.05 alpha level with two‐sided tests.

### 3.2 Twelve‐month treatment analysis

Twelve‐month prevalence of treatment was estimated for individual disorders within and across treatment sectors. We then examined treatment adequacy only within classes of disorder because of the sparseness of treatment data. Standard error (SE) of treatment prevalence estimates were obtained using the Taylor series linearization method (Wolter, 1985) implemented in SAS 9.4 software to adjust for the sample design and weighting of the sample. Logits and logits ± 2SE were exponentiated to produce ORs and 95% CIs. Multivariate significance tests of predictor sets were carried out using Wald χ2 calculated from Taylor series design based coefficient variance–covariance matrices. Statistical significance was evaluated consistently at the 0.05 level with two‐sided tests.

## 4 RESULTS

### 4.1 Twelve‐month prevalence and severity

The prevalence of any anxiety or mood disorder in the 12 months preceding the interview was 21.1% (Table 1) with an overall persistence rate of 74.7% (Table 1). Among those with any 12‐month disorder: 10.4% were considered mild, 38.7% were considered moderate, and 50.9% were considered severe cases (Table 1). Typically, any anxiety disorder (75.6%) was likely to persist more than any mood disorder (64.0%) with GAD among the highest (83.4%) and MDD (59.7%) among the lowest in persistence (Table 1).

**TABLE 1:** Twelve‐month prevalence and persistence of DSM‐5/CIDI disorders in the World Mental Health Qatar study.

Disorder	Prevalence		Persistence		Severity +					
					Mild		Moderate		Serious	
	%	SE	%	SE	%	SE	%	SE	%	SE
Anxiety										
Panic disorder^a^	2.0	0.2	78.8	3.7	6.2	2.5	26.7	5.2	67.1	5.2
Generalized anxiety disorder^a^	4.7	0.3	83.4	1.9	2.9	1.4	20.5	2.8	76.6	2.6
PTSD^b^	13.2	0.7	72.7	1.8	12.1	2.2	43.1	2.6	44.8	2.3
Obsessive compulsive disorder^b^	2.0	0.2	69.7	4.0	7.4	3.8	29.4	5.3	63.2	6.1
Any anxiety disorder^b^	16.2	0.8	75.6	1.7	12.1	1.8	41.7	2.2	46.1	2.0
Mood										
Major depressive disorder^a^	7.2	0.3	59.7	1.9	3.1	1.1	35.9	3.1	61.1	3.1
Bipolar I/II disorder^a^	3.9	0.3	74.0	2.6	0.0	0.0	0.0	0.0	100.0	0.0
Any mood disorder^a^	11.1	0.4	64.0	1.7	2.0	0.7	23.0	2.2	75.0	2.1
Any^b^	21.1	0.8	74.7	1.3	10.4	1.5	38.7	2.1	50.9	1.9
I. Distribution of the number of disorders by severity										
1 disorder^b^	13.5	0.7	‐‐‐	‐‐‐	98.3	0.8	78.6	2.1	45.8	2.4
2 disorders^b^	4.2	0.4	‐‐‐	‐‐‐	1.7	0.8	15.7	2.0	27.0	2.3
3+ disorders^b^	3.4	0.3	‐‐‐	‐‐‐	0.0	0.0	5.7	1.2	27.1	2.4
II. Distribution of severity by the number of disorders										
1 disorder^b^			‐‐‐	‐‐‐	16.0	2.3	47.6	2.9	36.5	2.4
2 disorders^b^			‐‐‐	‐‐‐	0.9	0.6	30.4	3.3	68.8	3.3
3+ disorders^b^			‐‐‐	‐‐‐	0.0	0.0	13.8	2.7	86.2	2.7
Any 12‐month disorder^b^	21.1	0.8	‐‐‐	‐‐‐	10.4	1.5	38.7	2.1	50.9	1.9

^a^ Part I weight.

^b^ Part II weight.

Related to persistence were severity and the number of disorders – both strongly related to each other in our sample. Among those who met the criteria for mild severity, the majority (98.3%) were participants with a single disorder, while among those with three or more disorders, the majority (86.2%) were participants with a severe condition (Table 1). Also shown in Table 1, respondents who had two comorbid disorders were nearly twice as likely to be rated serious as those with only one disorder (68.8 vs. 36.5%; χ^2^_1_ = 44.0, *p* < 0.001). And respondents with three or more 12‐month disorders were about 20% more likely than those with two disorders to be rated serious (86.2 vs. 68.8%; χ^2^_1_ = 5.2, *p* = 0.027).

### 4.2 Sociodemographic correlates of 12‐month disorders

Female relative to male gender was associated with both anxiety and mood disorders in the 12‐months preceding the interview and with their persistence (Table 2).

**TABLE 2:** Demographic predictors of 12‐month prevalence of DSM‐5/CIDI disorders and persistence among lifetime cases in the World Mental Health Qatar study.

	Any anxiety				Any mood				Any disorder			
	Prevalence (*N* = 2583, the entire part II sample)		Persistence (*N* = 883, all part II respondents with any anxiety disorder)		Prevalence (*N* = 5195, the entire part I sample)		Persistence (*N* = 883, all part I respondents with any mood disorder)		Prevalence (*N* = 2583, the entire part II sample)		Persistence (*N* = 1185, all part II respondents with any mental disorder)	
Predictor	OR	95% CI	OR	95% CI	OR	95% CI	OR	95% CI	OR	95% CI	OR	95% CI
Age												
18–29	1.54^a^	(1.01–2.35)	0.48	(0.22–1.05)	3.10^a^	(2.14–4.47)	1.4	(0.71–2.74)	2.01^a^	(1.34–3.00)	1.01	(0.54–1.86)
30–39	1.81^a^	(1.28–2.55)	0.66	(0.33–1.36)	3.08^a^	(2.16–4.39)	1.76^a^	(1.03–3.00)	2.18^a^	(1.55–3.08)	1.39	(0.80–2.41)
40–49	1.28	(0.91–1.80)	0.43^a^	(0.22–0.84)	1.89^a^	(1.29–2.77)	0.94	(0.52–1.69)	1.50^a^	(1.05–2.15)	0.82	(0.47–1.45)
Female	2.03^a^	(1.60–2.57)	1.90^a^	(1.23–2.94)	1.85^a^	(1.52–2.25)	1.45^a^	(1.11–1.88)	1.88^a^	(1.53–2.29)	1.55^a^	(1.08–2.23)
Education												
Student	2.47	(0.78–7.79)	^a^		0.76	(0.21–2.77)	0.46	(0.07–2.96)	1.53	(0.48–4.84)	0.45	(0.09–2.22)
Low education	1.23	(0.80–1.89)	3.52^a^	(1.30–9.55)	0.92	(0.61–1.38)	2.04^a^	(1.14–3.67)	1.3	(0.90–1.87)	3.54^a^	(1.84–6.80)
Low‐average	1.2	(0.91–1.59)	2.70^a^	(1.64–4.43)	0.95	(0.76–1.17)	1.35	(0.91–1.99)	1.05	(0.82–1.36)	2.17^a^	(1.38–3.41)
High‐average	1.08	(0.74–1.58)	1.74	(0.87–3.50)	1.08	(0.79–1.47)	1.32	(0.77–2.24)	1.01	(0.71–1.44)	1.57	(0.98–2.52)
Marital status												
Previously married	1.71^a^	(1.13–2.60)	1.81	(0.84–3.90)	2.50^a^	(1.83–3.40)	2.37^a^	(1.33–4.23)	1.70^a^	(1.18–2.44)	2.24^a^	(1.23–4.10)
Never married	1.28	(0.92–1.77)	1.55	(0.93–2.57)	1.45^a^	(1.15–1.83)	1.6	(0.98–2.61)	1.34	(0.98–1.83)	1.49	(0.88–2.52)

*Note*: Chi‐sq (*p*‐value)/chi‐sq degrees of freedom: Any anxiety prevalence: age = 4.0(0.013)/3, female = 36.2(<0.001)/1, education = 1.3(0.28)/4, marital status = 4.0(0.024)/2, model: 6.9(<0.001)/10. Any anxiety persistence: age = 2.8(0.05)/3, female = 8.7(0.005)/1, education = 6.9(<0.001)/3, marital status = 1.9(0.16)/2, model: 3.4(0.003)/9. Any mood prevalence: age = 16.3(<0.001)/3, female = 39.9(<0.001)/1, education = 0.2(0.94)/4, marital status = 20.5(<0.001)/2, model: 21.3(<0.001)/10. Any mood persistence: age = 3.6(0.019)/3, female = 7.9(0.007)/1, education = 2.2(0.08)/4, marital status = 5.6(0.006)/2, model: 5.4(<0.001)/10. Any disorder prevalence: age = 7.1(<0.001)/3, female = 39.8(<0.001)/1, education = 0.7(0.61)/4, marital status = 5.1(0.01)/2, model: 7.3(<0.001)/10. Any disorder persistence: age = 2.7(0.06)/3, female = 6.1(0.017)/1, education = 7.5(<0.001)/4, marital status = 3.6(0.035)/2, model: 6.2(<0.001)/10.

^a^ The predictor ‘Student’ was dropped as there are too few students in the persistence analysis of any anxiety disorders.

Relative to the 50+ years group, all younger age groups were associated with 12‐month mood and anxiety disorders except for the 40–49 years group, which did not differ from the 50+ years group in the 12‐month prevalence for any anxiety disorder. Only comparing 40–49 years relative to 50+ years significantly predicted less persistence for any anxiety disorder, while only comparing 30–39 years relative to 50+ years significantly predicted increased persistence of any mood disorder (Table 2).

Both previously and never married compared to currently married groups predicted any 12‐month mood disorder, but only previously married predicted 12‐month prevalence of any anxiety disorder compared to currently married group. Previously married significantly predicted persistence of any mood disorder, but neither previously married nor never married predicted persistence for any anxiety disorder relative to currently married group (Table 2).

Compared to bachelor's degree or higher (highest education category), all lower education levels were significantly associated with persistence, but consistently were not associated with the 12‐month prevalence of any anxiety or mood disorders. Compared to the highest education category, preparatory or secondary schooling significantly predicted persistence of any anxiety disorder, while only preparatory schooling or lower were significantly associated with persistence of any mood disorder relative to the highest education category (Table 2). Being a student was positively associated with any 12‐month anxiety, but negatively associated with any 12‐month mood disorder and its persistence. However, none of these findings were statistically significant and there were too few students in the persistence analysis of any anxiety disorder (Table 2).

### 4.3 Twelve‐month service use and treatment of disorders

#### 4.3.1 Prevalence of 12‐month treatment by disorder

The prevalence of overall 12‐month treatment among those with 12‐month mental disorder was only 15.8% (Table 3A) indicating that the majority (84.2%) of participants with a 12‐month mental disorder did not receive any treatment.

**TABLE 3:** Overall and proportional treatment among respondents 12‐months before the interview.

	No. of unweighted respondents	Mental health hospitalization		Mental health specialty		General medical		Any healthcare		Spiritual advisor		CAM		Any Non‐Healthcare^a^		Any treatment	
A. Overall treatment																	
Any anxiety disorder	675	1.7	(0.5)	7.9	(0.9)	2.8	(0.7)	9.4	(1.0)	6.2	(0.9)	4.1	(0.9)	9.5	(1.3)	16.5	(1.6)
Any mood disorder	531	2.0	(0.6)	8.5	(1.3)	3.1	(0.8)	10.6	(1.6)	7.2	(1.2)	2.8	(0.7)	9.1	(1.3)	17.7	(2.0)
Any disorder	890	1.4	(0.4)	7.4	(0.8)	2.6	(0.6)	9.0	(1.0)	6.0	(0.8)	3.2	(0.7)	8.6	(1.0)	15.8	(1.5)
No disorder	1693	0.1	(0.1)	1.1	(0.2)	0.5	(0.1)	1.5	(0.3)	1.2	(0.3)	1.1	(0.3)	2.3	(0.4)	3.6	(0.5)
Total part II sample	2583	0.4	(0.1)	2.4	(0.3)	1.0	(0.2)	3.1	(0.3)	2.2	(0.3)	1.6	(0.3)	3.6	(0.4)	6.1	(0.5)
B. Proportional treatment (among respondents with any 12‐month treatment)																	
Any anxiety disorder	125	10.2	(3.0)	47.9	(4.3)	17.0	(4.0)	56.7	(4.5)	37.4	(4.7)	24.7	(4.2)	57.8	(4.6)		
Any mood disorder	98	11.2	(3.1)	48.0	(4.6)	17.5	(4.4)	59.6	(4.9)	40.6	(4.7)	15.6	(3.6)	51.3	(4.9)		
Any disorder	155	8.8	(2.4)	47.0	(3.3)	16.6	(3.6)	57.0	(3.9)	37.8	(4.3)	20.3	(3.4)	54.6	(3.9)		
No disorder	90	3.3	(2.1)	30.5	(5.9)	15.3	(3.8)	40.9	(6.5)	33.3	(5.9)	31.6	(6.1)	63.1	(6.0)		
Total part II sample	245	6.3	(1.9)	39.5	(3.8)	16.0	(2.5)	49.6	(4.1)	35.7	(3.5)	25.5	(3.8)	58.5	(4.0)		

*Note*: Values are percent with standard errors in parenthesis.

^1^Part II weight.

^a^ Any non‐healthcare includes CAM (complimentary and alternative therapies) and counseling sessions with a spiritual advisor.

The distribution of overall 12‐month treatment across the different sectors within healthcare and non‐healthcare were very similar for any 12‐month anxiety or mood disorder (Table 3A). Among those with any 12‐month disorder, treatment occurred equally in healthcare and non‐health care settings at roughly 9% (Table 3A). Approximately 7.4% of those with a disorder received treatment from MHS, while 2.6% received treatment from GMP. Among those with a mental disorder who received treatment in a non‐healthcare setting, CAM constituted only 3.2%, while 6.0% received treatment from SA.

For those with no mental disorder (Table 3A), 3.6% overall received any treatment for mental health problems. Treatments within healthcare were 1.5% (1.1% of them receiving treatment from MHS while 0.5% received treatment from GMP) and 2.3% received treatment in a non‐healthcare setting. Also among those with no 12‐month disorder, CAM treatment constituted higher proportions of non‐healthcare treatments (1.1/2.3 = 48%) than those with a disorder (3.2/8.6 = 37%).

**FIGURE 1:**
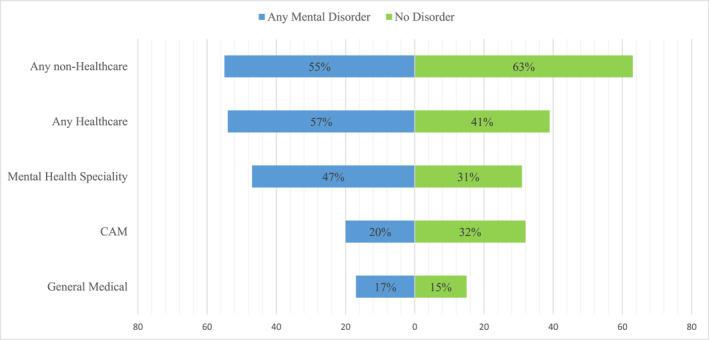
Proportional 12‐month treatment by healthcare and non‐healthcare sectors for those with any 12‐month mood or anxiety disorder.

Similar patterns as those described above were also observed in the proportional treatment data shown in Table 3B. The proportional 12‐month treatment among any 12‐month disorder was highest with a MHS (47%) followed by counselling sessions with a spiritual advisor (38%), CAM (20%), and lowest with a GMP (17%). As shown in Figure 1, equal treatments across health and non‐healthcare settings for those with any 12‐month disorder, but more non‐healthcare compared to healthcare treatments received for those with no disorder. Mental Health Specialists were frequented more by those with any disorder compared to those with no disorder, while similar proportions visited a GMP among those with and without a 12‐month mental disorder. Higher proportions of CAM treatments were found among those with no disorder compared to those with a disorder (Figure 1).

**FIGURE 2:**
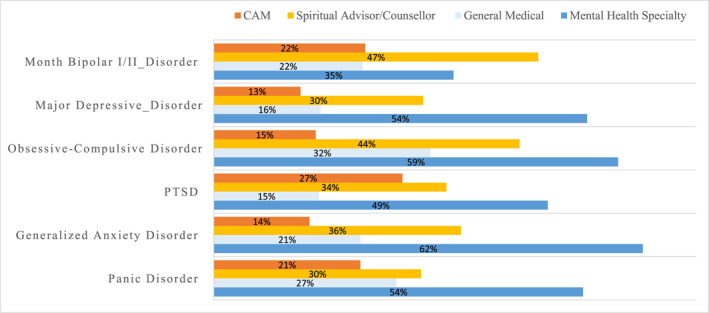
Proportional 12‐month treatment in each sector among respondents with specific 12‐month mental disorders who received any 12‐month treatment. The percentages add up to more than 100 percent because some people received treatments in different sectors.

The proportional 12‐month treatment across different sectors for the specific 12‐month disorders are also shown in Figure 2. All disorders had their highest proportional treatment among MHS with exception of BPD in which MHS treatment was second highest surpassed by treatment received spiritual advisors. Among those who received 12‐month MHS treatment, GAD had the highest treatment proportion of any 12‐month disorder (62%) followed by OCD (59%), PD and MDD (54%), PTSD (49%), and lowest for BPD at only 35% (Figure 2). Instead, BPD had the highest treatment from spiritual advisors at 47%, followed by OCD (44%), GAD (36%), PTSD (34%), and lowest for MDD and PD at 30% (Figure 2). Among those with 12‐month disorder, GMP treatment was highest for 12‐month OCD (32%), followed by PD (27%), BPD (22%), GAD (21%), MDD (16%), and lowest for PTSD (15%). Among those who received 12‐month treatment in the CAM sector, 12‐month PTSD had the highest proportional treatment at 27%, while MDD the lowest at 13% (Figure 2).

#### 4.3.2 Adequacy of 12‐month treatment by sector

Overall, 10.6% of those with any 12‐month disorder received 12‐month treatment that was considered minimally adequate regardless of the provider or sector from which they received treatment (Table 4). These estimates were the same for any 12‐month anxiety and any mood disorder roughly at 12% (Table 4). The corresponding estimate for proportional treatment within any sector for any 12‐month disorder was 67.2% (Table 4) with slightly higher estimate for 12‐month treatment for any 12‐month anxiety (69.8%) than any mood disorder (66.2%) within any sector. Adequacy of treatment was higher within healthcare (74.6%) compared to non‐healthcare (64.6%) sectors (Table 4). For healthcare sectors, adequacy was the same for 12‐month treatment within Mental Health Specialists (77.2%) and General Medical Professionals (76.5%), while it was highest for CAM (95.9%) within the non‐healthcare sector (Table 4).

**TABLE 4:** Adequacy of 12‐month treatment.^a^

	Mental health hospitalization		Mental health specialty		General medical		Any healthcare		Spiritual advisor		CAM		Any non‐healthcare		Any treatment	
	%	(SE)	%	(SE)	%	(SE)	%	(SE)	%	(SE)	%	(SE)	%	(SE)	%	(SE)
I. Minimally adequate treatment among those with any 12‐month disorder																
Anxiety	1.3	(0.4)	6.1	(0.9)	2.4	(0.6)	7.0	(1.1)	3.5	(0.8)	3.9	(0.9)	6.7	(1.2)	11.5	(1.5)
Mood	1.6	(0.5)	6.8	(1.2)	2.2	(0.6)	8.2	(1.3)	3.4	(0.7)	2.6	(0.7)	5.2	(1.0)	11.7	(1.5)
Any	1.1	(0.3)	5.7	(0.8)	2.0	(0.5)	6.7	(0.9)	3.1	(0.6)	3.1	(0.7)	5.6	(0.9)	10.6	(1.3)
II. Minimally adequate treatment among those with any 12‐month disorder and within treatment sector																
Anxiety	76.9	(12.0)	76.6	(6.6)	85.4	(7.1)	74.9	(6.3)	56.8	(8.3)	95.8	(3.1)	70.3	(5.9)	69.8	(4.9)
Mood	78.8	(13.2)	80.5	(6.0)	71.3	(10.4)	77.6	(5.2)	47.5	(8.3)	95.0	(4.9)	57.0	(7.9)	66.2	(4.9)
Any	78.4	(11.3)	77.2	(5.6)	76.5	(7.9)	74.6	(5.1)	51.1	(7.5)	95.9	(3.1)	64.6	(5.9)	67.2	(4.3)

^a^ Part II weight.

#### 4.3.3 Adequacy of 12‐month treatment by sector and disorder severity

As shown in Table 5, any 12‐month treatment varied by disorder severity – the more serious the disorder, the greater the percentage of treatment received (*χ* ^2^ = 7.24, *p* = 0.027): 19% for serious, 13.3% for moderate, and 9.5% for mild (Table 5). Within sectors, this association was significant for any treatments received by MHS (*χ* ^2^ = 7.44, *p* = 0.024), as well as within non‐healthcare sector (*χ* ^2^ = 7.77, *p* = 0.021). In terms of adequacy of treatment and disorder severity (Table 5), the most significant difference between serious, moderate and mild disorders was observed only within MHS sector (*χ* ^2^ = 21.42, *p* < 0.001).

**TABLE 5:** Twelve‐month treatment and minimally adequate treatment by severity of DSM‐5 disorders.^a^

	Serious (weighted *n* = 483)		Moderate (weighted *n* = 336)		Mild (weighted *n* = 71)		Any (weighted *n* = 890)		Difference between serious, moderate, and mild	
	%	(SE)	%	(SE)	%	(SE)	%	(SE)	χ^2^	*p*‐value
I. Any treatment										
General medical	3.4	(0.8)	2.2	(0.8)	0.8	(0.9)	2.6	(0.6)	2.85	0.241
Mental health specialty	10.4	(1.4)	5.4	(1.1)	5.3	(2.7)	8.0	(0.8)	7.44	0.024
Healthcare	11.3	(1.5)	6.8	(1.3)	6.1	(2.9)	9.0	(1.0)	5.60	0.061
Non‐healthcare	10.9	(1.6)	7.1	(1.6)	3.4	(1.7)	8.6	(1.0)	7.77	0.021
Any treatment	19.0	(2.0)	13.3	(2.1)	9.5	(3.8)	15.8	(1.5)	7.24	0.027
II. Minimally adequate treatment among those with any 12‐month disorder and any 12‐month treatment										
General medical	14.3	(4.0)	10.6	(4.8)	8.8	(8.7)	12.7	(3.2)	0.81	0.666
Mental health specialty	44.8	(5.3)	31.4	(6.2)	11.8	(11.3)	38.4	(3.9)	21.42	0.000
Healthcare	47.2	(5.5)	38.2	(6.8)	20.6	(13.9)	42.6	(4.3)	7.24	0.027
Non‐healthcare	36.5	(5.9)	34.1	(7.5)	30.0	(12.6)	35.3	(4.3)	0.74	0.689
Any treatment	68.6	(5.4)	67.8	(7.1)	50.5	(14.9)	67.2	(4.3)	2.50	0.286

*p < 0.05.

^a^ Part II weight.

## 5 DISCUSSION

Qatar is one of the eighteen countries in the EMR. Knowledge of the burden of mental disorder in the EMR to date mostly come from the Global Burden of Disease (GBD) 2013 study (Charara et al., 2017). The GBD study used synthetic prevalence data to estimate DALYs (disability‐adjusted life years) that capture both fatal and non‐fatal health outcomes by summing YLLs and YLDs. This study reported that mental disorders account for 5.6% of the global burden of disease in the EMR and that most DALYs in this region are due to depressive disorders followed by anxiety disorders (Charara et al., 2017). Furthermore, nearly all EMR countries had a higher mental disorder burden than the global burden of mental disorder (Charara et al., 2017). Moreover, this study pointed to a 10% increase in the burden of mental disorder in the region compared to earlier data from the 1990 GBD study (Charara et al., 2017). Authors attributed these findings to the low region's per capita expenditure and suboptimal mental health service provision relative to the global levels, which was pervasive across low‐, middle‐, and high‐income countries of the EMR (Charara et al., 2017). To date, only few countries in the EMR have collected non‐synthetic population‐based data as part of the WMH Survey Initiative. Our study is the first to collect such data in Qatar's general population as part of this global initiative. Here we summarize and discuss some of our main findings on the 12‐month prevalence and the sociodemographic correlates of common mental disorders and their treatment seeking in Qatar.

### 5.1 Twelve‐month prevalence & severity

Symptoms of mood and anxiety disorders among Qatar's Arab population are persistent. Just over 1 in 5 people had a DSM‐5 disorder in these categories in the 12 months preceding the interview. Approximately 3 in 4 of these cases persisted from some point prior to those 12 months. This means that the majority of people with a lifetime disorder also had a 12‐month disorder that was not resolved.

The majority of the cases with any 12‐month disorder were also moderate to severe: 39% of these cases being potentially serious, 51%, moderate, and only 10% were mild cases. Consistent with other studies within the WMH consortium (Kessler et al., 2009), the 12‐month prevalence of any mood or anxiety disorder in Qatar was marked by severity and multimorbidity. Both of these variables were strongly associated with each other in our study. For example, the majority (86.2%) of participants, meeting the criteria for three or more disorders in the 12‐month prior to the interview, also had a severe condition. Also, respondents with three or more 12‐month disorders were more likely to be rated serious than those with two disorders. Together, severity and multimorbidity together appear to compound severity contributing to persistence of mental disorders in Qatar's Arab population surveyed.

Similar patterns in lifetime prevalence and risk reported in this issue (Khaled, Alhussaini, Alabdulla, et al., 2024) were also observed for the 12‐month prevalence at the disorder class and the specific disorder level. Any anxiety disorder was higher in prevalence (16% vs. 11%) and more persistent (76% vs. 64%) than any mood disorder. However, higher percentage of mood disorders met the criteria for serious severity in the 12‐months preceding the interview compared to any anxiety (75% vs. 46%). Like lifetime prevalence and projected risk (Khaled, Alhussaini, Alabdulla, et al., 2024), the 12‐month prevalence was among the highest for PTSD (13.2%) and MDD (7.2%), while among the lowest (2.0%) for PD and OCD. PTSD also had relatively high persistence rate surpassed only by GAD, BPD, and PD, while the lowest persistence was among those with MDD despite its relatively higher 12‐month prevalence rate compared to other disorders.

Surpassed by the US (26%) (Kessler, 2005), the 12‐month prevalence of mental disorder in Qatar was comparable to Saudi Arabia (Altwaijri, Al‐Subaie, et al., 2020). Although relatively fewer disorders were assessed in Qatar than any other Arab country in the WMH consortium, Qatar's 12‐month prevalence estimate for any mood or anxiety disorder were among the highest in the region. Furthermore, Qatar had the highest proportions of serious disorder classification (50.9%) than any country in the region – surpassing Saudi Arabia (39.0%) (Altwaijri, 2020) and Lebanon (27.0%) (Karam et al., 2006), as well as developed countries like the US (22.3%) (Kessler et al., 2005). This high prevalence indicates a pressing need for further investigation and investment in mental health services. There are many causes for the high prevalence in Qatar, which may also include migration (Khaled, 2019; Khaled & Gray, 2019).

### 5.2 Sociodemographic correlates of twelve‐month disorders

Twelve‐month prevalence of any mood or anxiety disorder was associated with younger age (relative to 50+ years) at the time of the interview, female compared to male gender, and previously married relative to currently married status. Female gender and previously married status was associated with both prevalence and persistence of any 12‐month mood or anxiety disorder in Qatar.

Education was not associated with 12‐month prevalence, but secondary education or less relative to Bachelor's degree or higher was significantly associated with persistence of 12‐month mood or anxiety disorder.

The early onset suggest that the burden of these disorders is likely to last longer and be persistent in young females. Hence families and doctors need to be aware of the possibility of serious mental illness in these groups. The fact that prevalence was also associated with lower education means that public education programmes need to focus on reaching out to those vulnerable groups.

### 5.3 Twelve‐month service use & treatment

#### 5.3.1 Overall prevalence of 12‐month treatment

Qatar's overall 12‐month treatment prevalence was 6.1%. Only 15.8% of those with a disorder in the past 12 months received any treatment compared to 3.6% of those without any disorder. Similar treatment rates were reported in Saudi Arabia (Al‐Habeeb et al., 2020). Both of these Arabian Gulf countries' overall treatment rates were comparatively higher than those reported in Lebanon (4.4%) and in Iraq (2.2%). Populations in high‐income emerging economies like Qatar and Saudi Arabia continue to have much lower rates of treatment compared to other high‐income developed countries, for example, 24.2% in Japan (Nishi et al., 2019), 41.1% in the US (Wang et al., 2006), and as high as 62% in Belgium (Bruffaerts et al., 2003).

It is important to facilitate access to services as well as to improve treatment adequacy and quality of available treatments for common mental disorders (Jorm et al., 2017). Also important is to develop educational programmes to raise awareness of mental disorder and the need to seek treatment early. These initiatives that have already started in Qatar including the mental health helpline made available to the public during the COVID‐19 pandemic. Our data emphasizes that these programmes would benefit from expansion to high risk groups such as women and those less educated.

#### 5.3.2 Prevalence of 12‐month treatment by sector

In our study, the 12‐month proportional treatment rate among those with 12‐month disorder who are receiving any treatment (15.8%) was highest in the MHS (47.0%) sector, but comparatively lower in the GMP sector (16.6%). This is concerning given that GMP sector plays a pivotal role in gate‐keeping patients' access to specialized psychiatry care in (Wadoo et al., 2021). Since 2018, primary healthcare centers are mandated to implement national screening program for depression and anxiety with over 800K patients screened to date. However, the pathway‐to‐care for those who screen positive for these disorders remain in its infancy. As an emerging economy, one of the major challenges facing Qatar is staffing of mental health professionals like clinical psychologists and other allied mental health specialists. The majority of these MHS professionals are work‐migrants due to lack of credited educational/training programs, which are urgently needed for sustaining future's mental health workforce in the country.

Of importance is the spiritual advisors' role in the 12‐month treatment in the country. Religion plays an important role in the public as well as private life of most Qataris and Arab migrants. Additionally, religion does impact reported distress and help‐seeking for mental illness in Qatar (Khaled et al., 2022). Based on our data, seeking help from spiritual advisors and faith healers were common in the 12‐months preceding the interview among those who met the criteria for any anxiety or a mood disorder as well as those who did not. Previous studies from other Arab countries in the WMH consortium are also consistent with our findings. For policy makers in the country, the alignment of religious education with mental health promotion and treatment becomes an important area for future investigation and development.

#### 5.3.3 Prevalence of 12‐month treatment by disorder

In our study, 12‐month treatment was proportionally highest in the MHS with GAD as the leading treated disorder followed by OCD, MDD, and PD. Among the lowest treatment rates in the MHS sector was BPD, with a predominance of treatment for this disorder (47%) provided by SA and the lowest proportional treatment received equally in GM and CAM sectors. This is an important finding given that the most effective treatment for BPD is pharmacotherapy so people are not being treated by the service most appropriate for this disorder. This gap in service provision may have a bearing on the finding that the prevalence of BPD in Qatar is among the highest in the world. However, caution is needed in considering this possibility given that the CIDI clinical reappraisal study (Khaled, Amro, Abdelkader, et al., 2023) found that CIDI‐5 over‐diagnosed BPD in this survey.

Similar to BPD, PTSD was also among the disorders with the lowest proportional treatment in the MHS and in the GM sectors. This treatment gap may also explain the very high prevalence rates of PTSD in Qatar. Hence, future investigations into diagnosis and treatment seeking for BPD and PTSD are of importance to Qatar's mental health service.

#### 5.3.4 Adequacy of 12‐month treatment by sector & disorder severity

In Qatar, the prevalence of any 12‐month minimally adequate treatment received among those with any 12‐month mental disorder was low (approximately 11%). However, the proportion of those with 12‐month disorder who received treatment within any sector was roughly 67% with higher minimally adequate treatment received in the healthcare compared to non‐healthcare sectors (74.6% vs. 64.6%). The adequacy was the same for 12‐month treatment within MHS (77.2%) and GMP (76.5%), while it was highest for CAM (95.9%) within the non‐healthcare sector. In comparison to Qatar, approximately 14% of respondents with a 12‐month disorder received minimally adequate treatment in Saudi Arabia (Al‐Habeeb et al., 2020). In addition, the proportion of those with 12‐month disorder who received treatment within any sector was much lower in Saudi Arabia than Qatar at 45.2%. Moreover, the proportion of treatment received in the MHS was also much higher than GM sectors in Saudi Arabia at 68.9% and 43.5%, respectively (Al‐Habeeb et al., 2020). The general medical sector is one of the largest sources for mental health service delivery globally providing 70%–80% of all mental health services in most high‐income countries (Cia et al., 2019; Wang et al., 2007). Notably, adequacy of treatment within the healthcare system for both MHS and GM sectors in Qatar are well within the range reported for high‐income countries.

#### 5.3.5 Adequacy of 12‐month treatment by sector & disorder severity

Overall, serious mood or anxiety disorders were significantly more likely to receive 12‐month treatment than mild or moderate disorders in Qatar. However, serious relative to mild or moderate disorders were significantly more likely to receive adequate treatment only within the MHS sector. In Saudi Arabia, in those with a 12‐month diagnosis in the MHS or GM, severity was not significantly associated with receiving adequate treatment except in the non‐healthcare sectors (Al‐Habeeb et al., 2020).

#### 5.3.6 Strengths and limitations

As the first population‐based epidemiological study on common mental disorders in Qatar this work highlights the extent to which common mental disorders affect that population. Our data also reveals that symptoms in this non‐clinical population can emerge and persist and hence have the potential to develop into more severe and persistent clinical illness. Hence, it is important for this data to inform public health strategies accordingly.

We only interviewed Arabs – Qatari and non‐Qatari residents, and a large proportion of Qatar's population is non‐Arabic. Practical constraints meant that not all mental disorders could be investigated (including substance use disorder). Like any such study it relied upon people's willingness to participate and declare their symptoms. Migrant status was an important correlate of lifetime prevalence, but was not included as a correlate in our findings of 12‐month prevalence. The work is an early exploration of prevalence, and more work is needed to understand the detail behind risk factors for specific mental disorders that affect Qatar and the Middle East more generally.

## 6 CONCLUSIONS

For any service planning, identifying correlates of prevalence and persistence are crucial. People with multiple disorders are particularly vulnerable and healthcare workers need to be vigilant to mental symptoms in this group. Anxiety, although common, in severe cases places a significant burden on people and tends to persist. As a result, early treatment of anxiety disorders is imperative. Here younger people, females, and those previously married are also vulnerable and so are people with lower education. In relation to services, on one hand, the mental health service in Qatar has low overall 12‐month treatment rates that are comparable to estimates reported to high‐income countries in the region (e.g. Saudi Arabia), but far lower than reported in high‐income countries in Europe and US. Additionally, far less people with any disorder in Qatar are receiving any care for mental health problems in the GMP sector relative to other sectors than reported in other high‐income countries that carried out WMH surveys. On the other hand, adequacy of treatment within the healthcare system for both MHS and GM sectors in Qatar are well within the range reported for high‐income countries. These are some encouraging signs in favor of the progress made by the mental health service in Qatar thus far. An important future direction is to improve treatment adequacy for mild and moderate disorders as currently only serious disorders are more likely to receive adequate treatment within the mental health service in the country, but that is not the case for mild or moderate disorders. That a large proportion of the population with symptoms lasting longer than 12 months had not received any treatment indicates either a reluctance to seek help or that help is not reaching out to those in need. There is therefore a pressing need for population‐wide educational programmes to help de‐stigmatise mental illness as well as make mental health services more accessible and effective.

## AUTHOR CONTRIBUTIONS

**Salma M. Khaled**: Conceptualization; data curation; formal analysis; writing ‐ original draft; methodology; investigation; supervision; project administration; writing ‐ review & editing; software; funding acquisition; resources. **Sheik Mohammed Al‐Thani**: Resources; writing ‐ review & editing. **Nancy A. Sampson**: Conceptualization; formal analysis; writing ‐ original draft; writing ‐ review & editing; validation; project administration; methodology. **Ronald C. Kessler**: Conceptualization; project administration; supervision; methodology; writing – original draft; writing ‐ review & editing; validation. **Peter W. Woodruff**: Conceptualization; writing ‐ original draft; writing – review & editing; methodology; funding acquisition. **Majid Alabdulla**: Conceptualization; writing ‐ review & editing; project administration; investigation; funding acquisition.

## CONFLICT OF INTEREST STATEMENT

In the past 3 years, Dr. Kessler was a consultant for Cambridge Health Alliance, Canandaigua VA Medical Center, Holmusk, Partners Healthcare, Inc., RallyPoint Networks, Inc., and Sage Therapeutics. He has stock options in Cerebral Inc., Mirah, PYM, Roga Sciences and Verisense Health.

## ETHICS STATEMENT

Qatar University (QU‐IRB 1219‐EA/20) approved the study. The study's goal and methods were verbally explained to participants. Before each survey interview, consent to participate were verbally obtained using a phone script. All data were encrypted and saved on Qatar University's secure server. Each participant was assigned a case number and individual identifiers were retained in a password‐protected folder only available to the lead principle investigator, senior research assistant, and data analyst. All study researchers, including interviewers, signed confidentiality agreements preventing the sharing or use of participant personal information.

## Data Availability

The data that support the findings of this study are available from Dr. Salma M. Khaled, the principal investigator of the study at skhaled@qu.edu.qa, upon reasonable request and pending additional ethical approval.
